# Epidemiological and clinical characteristics of older adults with burns: a 15-year retrospective analysis of 2554 cases in Wuhan Institute of Burns

**DOI:** 10.1186/s12877-023-03883-5

**Published:** 2023-03-22

**Authors:** Hong Wu, Maomao Xi, Weiguo Xie

**Affiliations:** Wuhan Institute of Burns, Tongren Hospital of Wuhan University (Wuhan Third Hospital), 241# Peng Liuyang Road, Wuhan, 430060 Wuchang District China

**Keywords:** Geriatrics, Burns, Epidemiology, Mortality, Risk factors

## Abstract

**Background:**

With the increase of geriatric burns, it’s urgent to summarize its characteristics. The aim of this study was to analyze the epidemiological and clinical characteristics of older adults with burns in a large center, and to provide suggestions for the prevention and treatment of geriatric burns.

**Methods:**

This retrospective study was conducted at Wuhan Institute of Burns which is the largest burn center in central China between 2004 to 2018. Demographic and clinical data of the 60 years or above older burn inpatients were collected from medical records, analyzed and compared among groups.

**Results:**

This study analyzed 2554 elderly burns, which included 50.9% in young geriatric group (60–69 years old), 32.9% in middle geriatric group (70–79 years old) and 16.2% in the oldest geriatric group (80 years old or above). The most common causes of elderly burns were flames (1081, 42.3%) and scalding (1041, 40.8%). Elderly burns with total body surface area (TBSA) of 0–9% accounted for 60.6% and the larger TBSA, the fewer number of patients. The majority of patients (70.5%) injured at home.The median of time interval from injury to admission was 7 h and the oldest geriatric group (24 h) was highest. One hundred and twenty-one cases (8.5%) were treated by cooling treatment, and 72.7% of these patients were treated less than 10 min. The median number of pre-injury diseases was one. Ninety patients (6.3%) had inhalation injury.The median length of stay (LOS) was 14 days.The median hospital cost was 10,410 CNY or 2137 CNY per % TBSA, which was correlated with TBSA, LOS, surgery, inhalation injury, number of pre-injury diseases and etiology. The mortality rate was 3.0% and correlated with TBSA, inhalation injury, pulmonary disease and Alzheimer’s disease. The lethal area 50% (LA_50_) for total admitted elderly burns was 78.3% TBSA (95% confidence interval [CI] = 69.8 ~ 89.9% TBSA).

**Conclusion:**

Geriatric burns was still common and even increasing in central China, with flame burns and scalds the most common causes, majority of whom injured at home and often had problems such as few cooling treatment, improper emergency management and delayed admission. TBSA, etiology, pre-injury diseases and inhalation injury were the risk factors of length of stay, hospital cost and treatment outcomes.

## Background

The world's population is ageing, with almost every country having an increasing number and proportion of older people in its population. Globally, the population aged 65 or over is growing faster than all other age groups. World Population Prospects 2022 states that the share of global population at ages 65 or above is projected to rise from 10% in 2022 to 16% in 2050 [1]. As the most populous developing country, China is also facing the problem of population aging. Since China entered the aging society in 2000, the proportion of the elderly population has increased by 8.4% in the last two decades, and the total number of elderly people 60 or above is 264 million, accounting for 18.7% of the total population according to the results of the seventh National Population Census of the Chinese mainland [2]. Wuhan is the largest city in central China, with a permanent population of 12.326 million, and also faces the problem of population aging [3].

With the increase of the global population aging, the problem of burns among the older adults needs urgent attention. Elderly burns often suffer from a variety of chronic diseases, and aging leads to thinning skin, reduced sensory abilities, and reduced ability to care for themselves [4–6]. Burns caused by cooking, bathing, and heating at home are more common in the elderly [7, 8], and the ability of the elderly to save themselves is limited. At the same time, it is also difficult to treat patients due to various diseases and advanced age [4, 5].

Most researches on elderly burns were mainly from developed countries such as the United States [9–11], the United Kingdom [12], the Netherlands [13], and Finland [14]. A few reports on elderly burns in China were from east coastal city Shanghai [15], south-west cities of Chongqing [5] and Chengdu [7]. As a vast and diverse territory and the most populous country in the world, there has been no report on elderly burns in central China. Wuhan is the biggest city in central China with more than ten million population, located in an area with hundreds of lakes and the surrounding hilly and mountainous, the climate humid and hot in summer and cold in winter. People in the area like to drink hot soup and soak feet with hot water, and in mountain areas coal and firewood still used for cooking and heating. Because of the geographical, climatological and social habitual reasons, burn incidence in Wuhan and surrounding areas are not only high but also characteristic, especially for elderly people. Wuhan Institute of Burns is one of the top burn centers in China [16], with 150 to 200 in inpatient beds and an annul admissions of over 5000 from Wuhan and surrounding areas [17]. This study retrospectively collected medical records of burn inpatients for 15 years and analyzed the detailed rich data in order to provide a reference for the prevention and treatment of older adults burns.

## Methods

### Patient inclusion and exclusion criteria

This retrospective study included burn patients aged 60 or older who were admitted to the burn center at Tongren Hospital of Wuhan University (Wuhan Third Hospital) between January 2004 and December 2018. Elderly burns in this study included flame burns, scalds, electrical contact burns, electrical flash burns, low-thermal-burns, contact burns with hot objects, medical-related burns, chemical burns, thermal crush injury and excluded patients with frostbite, radiation ulcers, and the purpose of treating chronic wounds, scars, and plastic surgery, etc. Ethical approval for data collection was granted by the Human Research Ethics Committee of the Tongren Hospital of Wuhan University (Wuhan Third Hospital) (No. KY2018-030).

### Protocol of treatment

After admitted, the patients were immediately checked, monitored and evaluated. The venous pathway was established for fluid resuscitation and anti-shock treatment [18]. Patients were resuscitated according to the formula of fluid infusion in shock phase of the Third Military Medical University. During fluid resuscitation, patient’s fluid infusion plan timely adjusted according to the patient's vital signs and monitoring indicators, and timely corrected the acid–base imbalance and water-electrolyte imbalance [19]. In addition, nasogastric tube was inserted early for patients with large area burns, and gastrointestinal decompression or early nutrition support was performed if necessary. Tracheotomy and mechanical ventilation were performed for patients with respiratory tract burns and large area burns. Continuous renal replacement therapy was performed for patients with severe water-electrolyte imbalance, chemical toxin absorption or severe renal dysfunction. Some annular full-thickness wounds were treated incision and tension reduction of burn eschar. For small area of superficial partial-thickness burn, the wounds were cleaned with 0.9% saline solution and 0.5% iodophor and dressed with Vaseline and dry gauzes. The dressings were changed in time according to the seepage situation, generally once a day. What’s more, xenograft coverage for large area of superficial or deep partial-thickness burns was performed in two days post-burn and escharectomy and wound coverage for extensive deep partial- and full-thickness burns by large sheet of allografts with auto-microskins was performed timely [20].

### Data collection and grouping definition

The following data were collected from the medical records: age, gender, etiology of injuries, admission date, total burn surface area (TBSA), depth of burn, location of accident, pre-hospital emergency management, time interval between injury and admission, pre-injury disease, complications, surgery, surgery cost, length of hospital stay (LOS), hospital cost, outcomes.

Patients were grouped by age as young geriatric group (60–69 years), middle geriatric group (70–79 years), and the oldest geriatric group (≥ 80 years). Patients were grouped by TBSA as small area (< 10% TBSA), medium area (10–29% TBSA), large area (30–49% TBSA) and extra-large area (≥ 50% TBSA). Pre-injury disease was defined as a chronic organ disease diagnosed and treated prior to the burn.The treatment outcomes were grouped as cured (when being discharged all wounds healed or only with scattered wounds which could be cured spontaneously), and death. In addition to treatment outcomes of cured and death, there were also patients who were not cured or gave up treatment. Patients who were not cured referred to those patients who were transferred to outpatient or other hospitals for treatment and patients who gave up treatment referred to patients who had received treatment for a very short time, generally less than 3 days, and then died at home. Different causes of burns were classified and defined, among which the low-thermal-burns referred to burns caused by low heat, such as hot water bag burns, infrared thermotherapy instrument burns. The medical-related burns referred to burns caused by medical-related behaviors, such as moxibustion-induced burns. Besides, the thermal crush injury referred to burns caused by heated mechanical pressure that results in burns in localized tissues and the potential damage to expose deeper tissues, and more common in industrial settings.

### Statistical analysis

SPSS22.0 and GraphPad Prism 6 were used for data analysis and graphing. Patients whose data for the above items were missed or unclear in their medical records were excluded from the item analysis. Continuous variables were tested for normality by Kolmogorov–Smirnov test, and then presented by mean ± standard deviation or median (25-75th percentile, Q1-Q3). Non-parametric test (Mann–Whitney U test) was used for comparison between the two groups, and analysis of variance and Kruskal Wallis test were used for comparison among multiple groups. Spearman rank correlation test was used for bivariate correlation test. The categorical variables were presented in terms of number of cases and percentage, and comparisons between groups were performed using Chi-squared test or Fisher exact probability method. Bonferroni correction was applied to the *P* values for multiple comparisons between groups. Multivariate logistic regression and multivariate linear regression were used for multivariate analysis. The lethal area 50 (LA_50_), a measure of burn survivability for the TBSA at which there will be 50% mortality, was calculated using probit analysis. Statistical significance was considered at a probability of *P* value < 0.05.

## Results

### Demographic characteristics

**Fig. 1:**
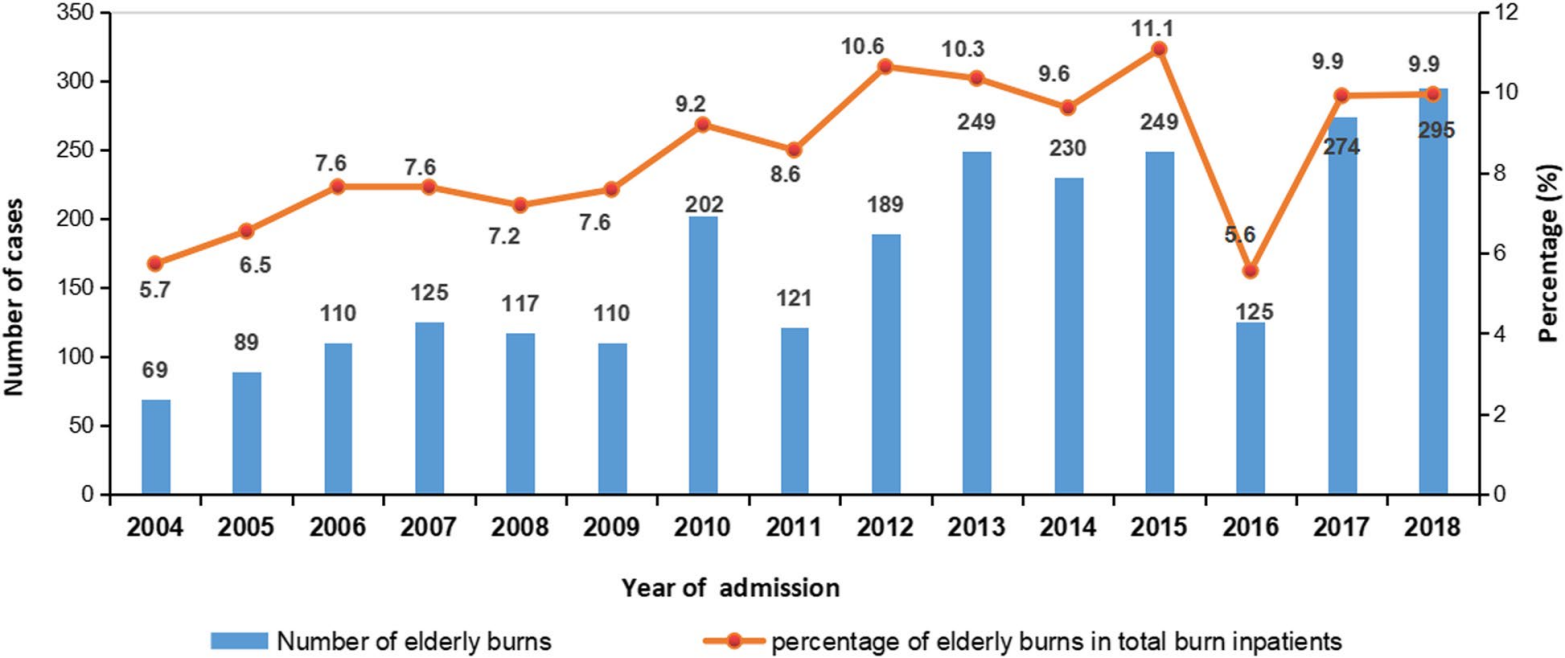
Number of elderly burn inpatients and their percentage in total burn inpatients from 2004 to 2018

**Fig. 2:**
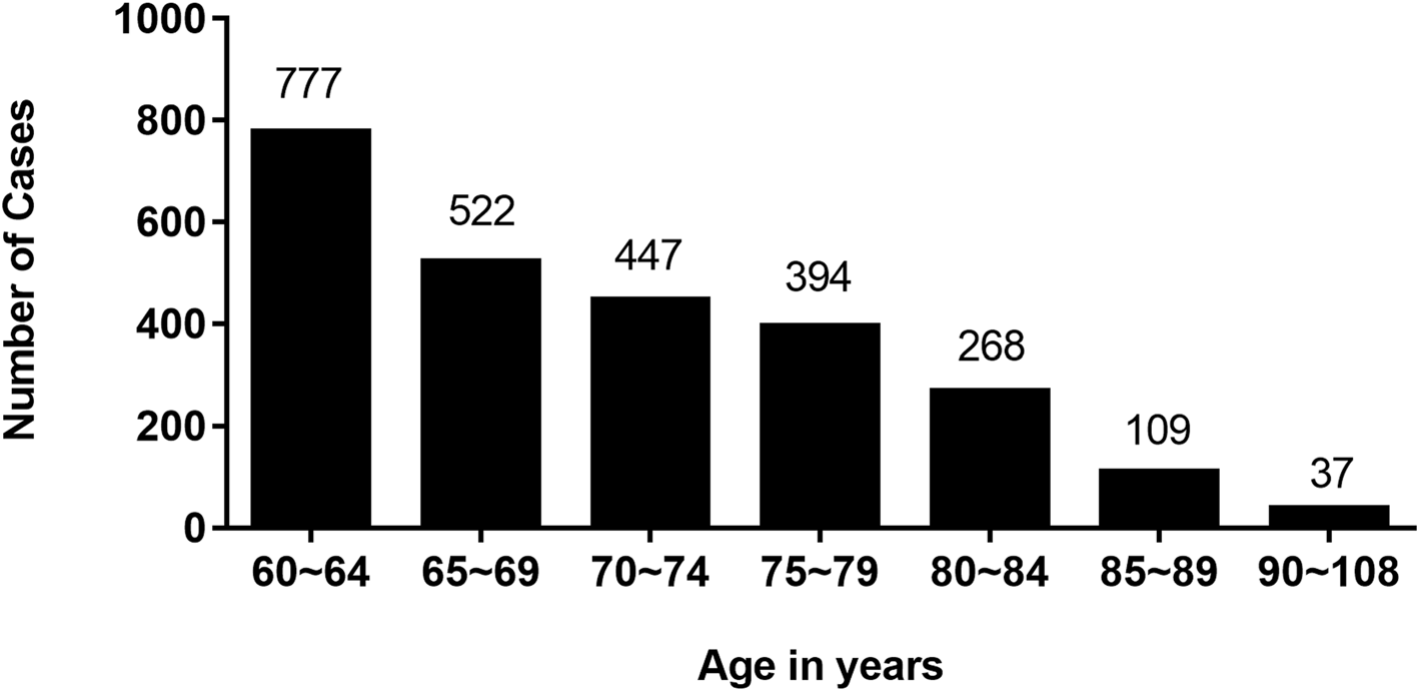
Age distribution of the elderly burn inpatients

This retrospective study included 2554 burn patients aged 60 years and older among 29,151 burn patients admitted to the burn center of Tongren Hospital of Wuhan University (Wuhan Third Hospital) between January 2004 and December 2018. In addition to the total number of burn inpatients affected by the refurbishment and relocation of wards in 2009, 2011 and 2016, there has been a gradual increase in the number of elderly burns over the past 15 years. Moreover, the percentage of elderly burns in total burn patients increased from 5.7% in 2004 to 9.9% in 2018 (Fig. 1). The average age of elderly burns was 70.5 ± 8.1 years, including young geriatric group (1299 cases, 50.9%), middle geriatric group (841 cases, 32.9%), and oldest geriatric group (414 cases, 16.2%). The number of elderly burns decreased with increasing age (Fig. 2). There were 1385 males (54.2%) and 1169 females (45.8%), with a male: female ratio of 1.2:1.

### Etiology

**Fig. 3:**
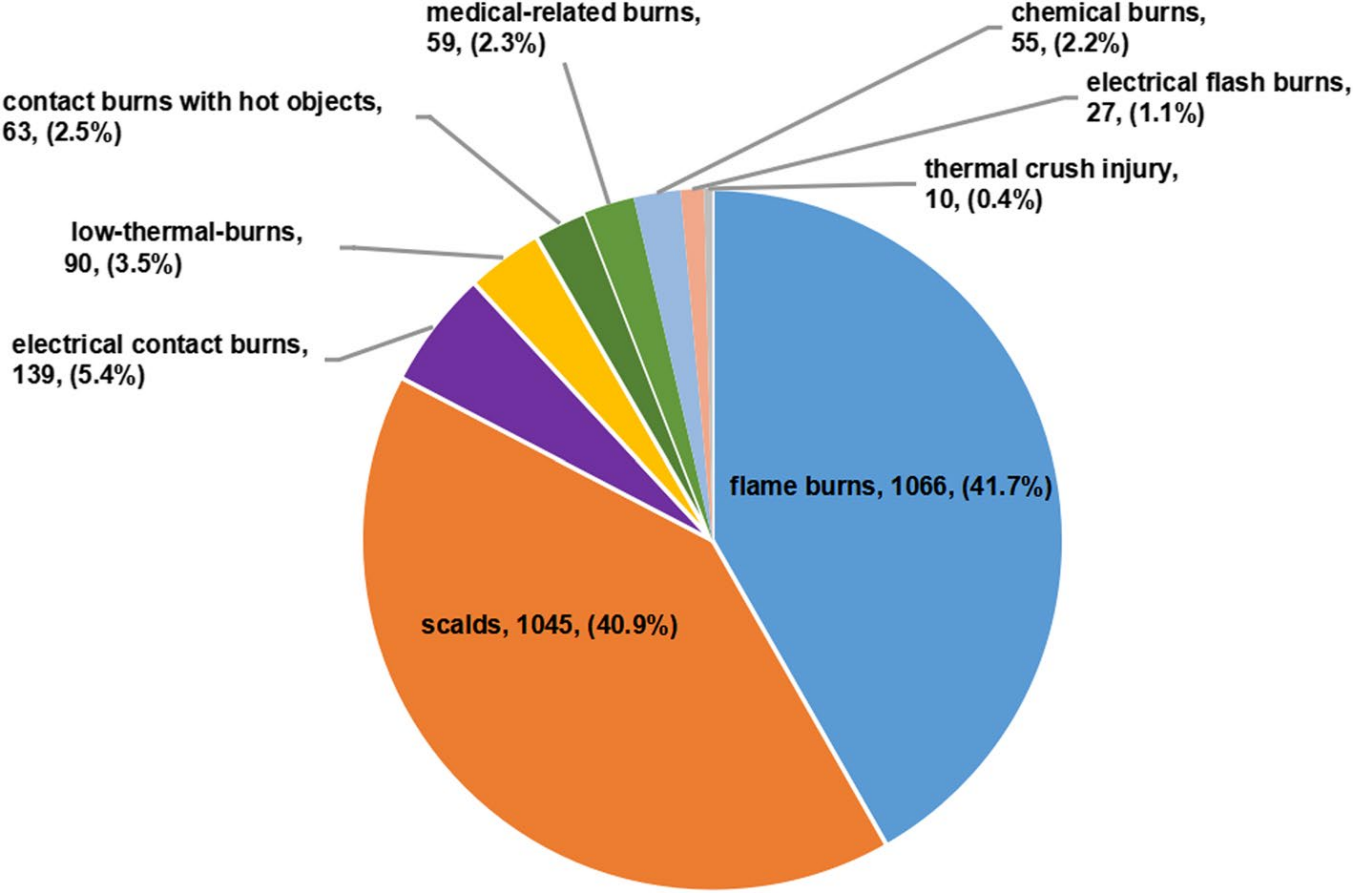
Percentages of different etiologies of the elderly burn inpatients

Among the elderly burns in this study, flame burns were the most common (1066 cases, 41.7%), followed by scalds (1045 cases, 40.9%), electrical contact burns (139 cases, 5.4%), low-thermal-burns (90 cases, 3.5%), contact burns with hot objects (63 cases, 2.5%), medical-related burns (59 cases, 2.3%), chemical burns (55 cases, 2.2%), electrical flash burns (27 cases, 1.1%), thermal crush injury (10 cases, 0.4%) (Fig. 3). The age difference of elderly burns with different etiologies was statistically significant (*F* = 10.696, *P* < 0.001).The average age of each etiology from high to low was: 73.6 ± 8.7 years old for low-thermal-burns, 73.5 ± 8.2 years old for thermal crush injury, 71.1 ± 8.3 years old for scalds, 71.7 ± 8.2 years old for contact burns with hot objects, 70.4 ± 8.0 years old for flame burns, 70.6 ± 6.5 years old for medical-related burns, 67.1 ± 7.2 years for chemical burns, 66.4 ± 6.0 years for electrical contact burns, and 64.2 ± 3.9 years for electrical flash burns. The average age of elderly burns with electrical contact burns or electrical flash burns was significantly lower than the average age of patients with low-thermal-burns, scalds and flames, respectively (*P* < 0.05). There was a significant difference in the male:female ratio for different etiologies (*x*^2^ = 148.959, *P* < 0.001). Among them, the male:female ratio of electrical contact burns was the highest (4.1:1), followed by thermal crush injury (2.3:1), contact burns with hot objects (2.0:1), flame burns (1.5:1), chemical burns (1.3:1), low-thermal-burns (1.1:1), medical-related burns (1.1:1), and scalds (0.8:1), and the percentage of male with electrical contact burns was significantly higher than that of male with other etiologies (*P* < 0.001) (Table 1).

**Table 1:** Differences in age and gender ratio for different etiologies of elderly burn inpatients

Etiology	N (%)	Age (y)	Male/female ratio
Flame burns	1066 (41.7)	70.4 ± 8.0	1.5
Scalds	1045 (40.9)	71.1 ± 8.3	0.8
Electrical contact burns	139 (5.4)	66.4 ± 6.0	4.1
Low-thermal-burns	90 (3.5)	73.6 ± 8.7	1.1
Contact burns with hot objects	63 (2.5)	71.7 ± 8.2	2.0
Medical-related burns	59 (2.3)	70.6 ± 6.5	1.1
Chemical burns	55 (2.2)	67.1 ± 7.2	1.3
Electrical flash burns	27 (1.1)	64.2 ± 3.9	/
Thermal crush injury	10 (0.4)	73.5 ± 8.2	2.3
Total	2554 (100.0)	70.5 ± 8.1	1.2
*x*^*2*^*/F*	-	10.696	148.959
*P* value	-	< 0.001	< 0.001

*TBSA* Total burn surface area, *LOS* Length of stay. Male/female ratio of electrical flash burns could not estimate because there was no female patients caused by this etiology

### Season of injury

Season of injury data was available in 2448 patients (95.8% of the total elderly burns), and most cases of elderly burns were in winter (December to February, 693 cases, 28.3%), followed by summer (June to August, 665 cases, 27.2%), spring (March to May, 609 cases, 24.9%), and autumn (September to November, 481 cases, 19.6%). Different etiologies tended to have significantly different seasonal distribution (*P* < 0.001), among which low-thermal-burns, contact burns with hot objects, and flame burns mostly occurred in winter (51 cases, 60.7%; 21 cases, 33.3%; 310 cases, 30.5%, respectively), hot crush burns, chemical burns, and scalds mostly occurred in spring (3 cases, 42.9%; 20 cases, 38.5%; 279 cases, 27.7%, respectively), and electrical contact burns and electrical flash burns mostly occurred in summer (62 cases, 45.9%; 9 cases, 36.0%, respectively).

### Total burn surface area (TBSA) and depth of burn

**Fig. 4:**
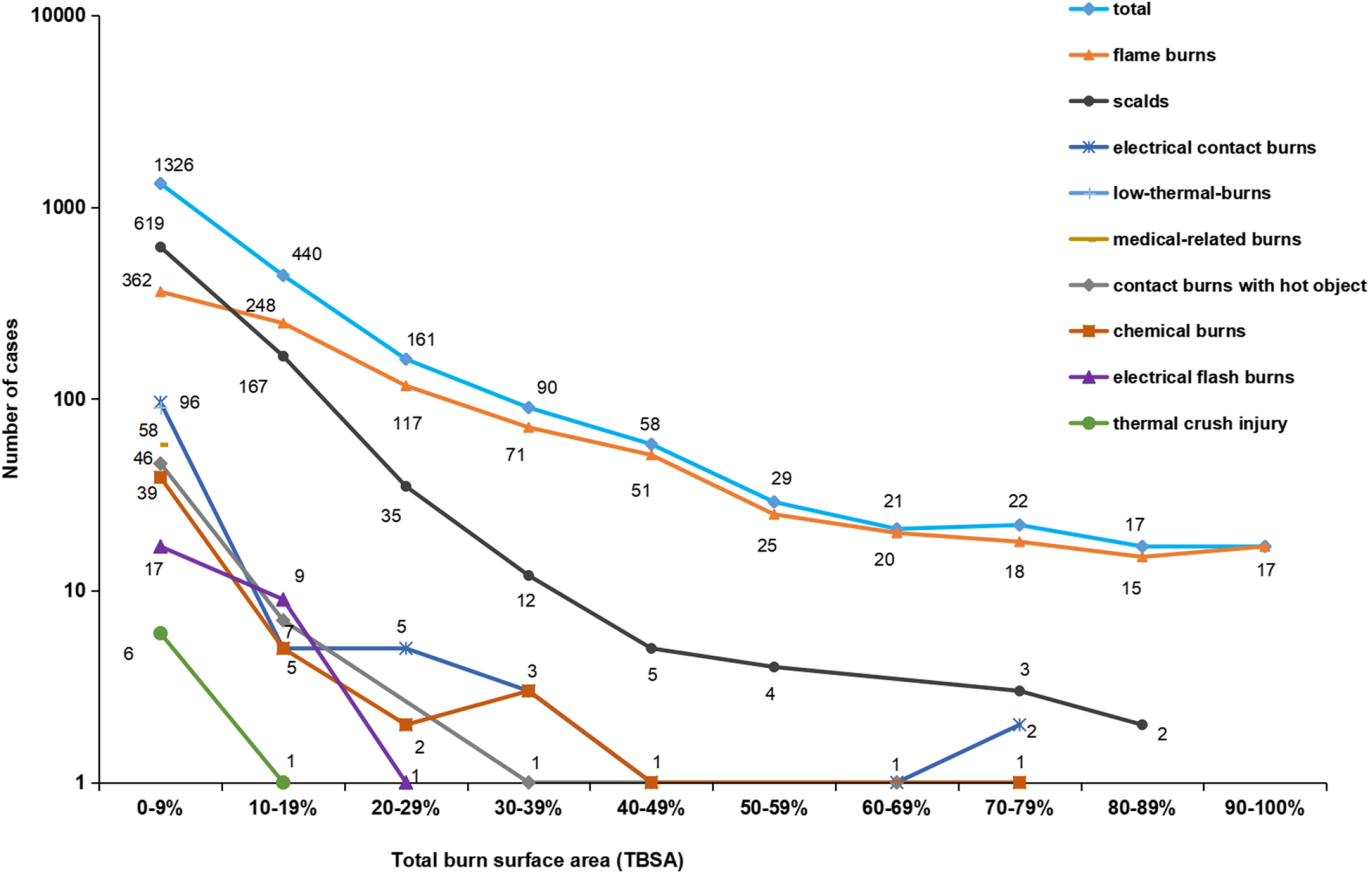
Distribution of TBSA in different etiologies of the elderly burn inpatients

Accurate TBSA were available for 2208 patients (86.5% of the total elderly burns). The median TBSA was 7% (25-75th percentile, Q1-Q3, 2%-15%) and ranged from 0.1–99.0%TBSA. In addition, depth of burn data were available in 1430 patients (56.0% of the total elderly burns). Regarding the maximum burn depth, most patients had full-thickness burns (65.5%, 937 cases) and the median full-thickness burn area was 0.7% (Q1-Q3, 0%-3%). The larger the burn area, the smaller the number of elderly burns (Fig. 4). The results revealed that most elderly burns (60.6%) were in small area group. There was a significant difference in the depth of burns among different etiologies (*x*^*2*^ = 167.693, *P* < 0.001). The percentage of elderly burns with full-thickness burns from high to low was electrical contact burns (100.0%), low-thermal-burns (97.6%), medical-related burns (96.6%), contact burns by hot objects (93.5%), chemical burns (77.1%), flame burns (63.7%), scalds (52.1%), and electrical flash burns (36.0%).

### Location of accident

Location of accident data were available in 1150 patients (45.0% of the total elderly burns), and the percentage of the location of accident from high to low was 70.5% (811 cases) at home, 24.7% (248 cases) in outdoors, 3.0% (35 cases) in the workplace, and 1.7% (20 cases) in other places such as hospitals or old age homes.

In the comparison of location of accident of elderly burns in different age groups, there was a significant difference in the percentage of injuries occurred at home (*x*^*2*^ = 22.945, *P* < 0.001).The results showed that older people were more likely to be injured at home. The proportions of elderly burns injured at home were 64.7% (366 cases) in the young geriatric group, 73.3% (283 cases) in the middle elderly group and 81.8% (162 cases) in the oldest geriatric group, respectively (Oldest geriatric group vs. young geriatric group: *P* < 0.001; middle geriatric group vs. young geriatric group: *P* = 0.015).

### Pre-hospital emergency management

Accurate pre-hospital emergency management data were available for 1420 patients (55.6% of the total elderly burns). There were 1113 cases (78.4%) did not do any pre-hospital emergency management after burn, 158 cases (11.1%) underwent self-treatment (such as scald cream, alcohol or iodophor, etc.), 47 cases (3.3%) used some folk remedies (such as toothpaste, eggs, soy sauce, vinegar, salt, etc.). In addition, there were 121 cases (8.5%) performed the cooling treatment, but most of them (88 cases, 72.7%) did it for less than 10 min.

Among elderly burn patients of different age groups, there was a statistically significant difference in the proportion of self-treatment after burn (*x*^*2*^ = 10.527, *P* = 0.005). In the elderly burns, the older they were, the more they did self-treatment. Moreover, the proportion of self-treatment in the young geriatric group, the middle geriatric group and the oldest geriatric group were 8.2%, 9.7% and 15.5%, respectively (Oldest geriatric group vs. young geriatric group: *P* = 0.003) (Table 2).

**Table 2:** Comparison of pre-hospital emergency management stratified per age category

Age categories(years)	Total (*N* = 1420)	60–69 (*n* = 791)	70–79 (*n* = 403)	80+ (*n* = 226)	*x*^*2*^	*P* value
**Without any emergency management**					2.686	0.261
Yes	1113 (78.4%)	624 (78.9%)	321 (79.7%)	168 (74.3%)		
No	307 (21.6%)	167 (21.1%)	82 (20.3%)	58 (25.7%)		
**Cooling treatment**					2.709	0.258
Yes	121 (8.5%)	76 (9.6%)	29 (7.2%)	16 (7.1%)		
No	1299 (91.5%)	715 (90.4%)	374 (92.8%)	210 (92.9%)		
**Duration of cooling treatment**					0.691	0.708
< 10 min	78 (64.5%)	51 (67.1%)	17 (58.6%)	10 (62.5%)		
≥ 10 min	43 (35.5%)	25 (32.9%)	12 (41.4%)	6 (37.5%)		
**Self-treatment**					10.527	0.005
Yes	139 (9.8%)	65 (8.2%)	39 (9.7%)	35 (15.5%)		
No	1281 (90.2%)	726 (91.8%)	364 (90.3%)	190 (84.5%)		
**Hospital consultation within 24 h**					5.699	0.058
Yes	890 (62.7%)	513 (64.9%)	249 (61.8%)	128 (56.4%)		
No	530 (37.3%)	277 (35.1%)	154 (38.2%)	99 (43.6%)		

There were 1420 patients (55.6% of the total elderly burns) who the time interval between injury and admission, the median of time interval between injury and admission was 7 (Q1-Q3, 2–144) hours, and 890 cases (62.7%) were admitted within 24 h after burn. There was a significant difference in the time interval between injury and admission after burn among different age groups (*x*^*2*^ = 6.026, *P* = 0.049). And, the median of the time interval between injury and admission in the young geriatric group, the middle geriatric group and the oldest geriatric group was 6 (Q1-Q3, 2–120) hours, 8 (Q1-Q3, 2–168) hours, and 24 (Q1-Q3, 2–192) hours, respectively (Oldest geriatric group vs. young geriatric group: *Z* = 2.356, *P* = 0.018).

### Pre-injury diseases and complications

There were 1429 patients (56.0% of the total elderly burns) who had pre-injury diseases information, the average number of pre-injury diseases in these elderly burns was 1.0 ± 1.1, and median of it was 1 (Q1-Q3,0–2). There were 635 cases had no pre-injury diseases (44.4%), 407 cases (28.5%) had one kind of pre-injury disease, 237 cases (16.6%) had two kinds of pre-injury diseases, 118 cases (8.3%) had three kinds of pre-injury diseases, 28 cases (1.1%) had four kinds of pre-injury diseases, 3 cases (0.2%) had five kinds of pre-injury diseases, and one case (0.1%) had 6 kinds of pre-injury diseases.

The number and percentage of pre-injury diseases of elderly burns from high to low was: 512 cases (35.8%) with hypertension, 199 cases (13.9%) with diabetes, 170 cases (11.9%) with heart disease, 123 cases (8.6%) with cerebral infarction, 51 cases (2.0%) with pulmonary disease, 43 cases (3.0%) with digestive tract disease, 30 cases (2.1%) with Alzheimer’s disease, 23 cases (1.6%) with epilepsy, 14 cases (1.0%) with cancer and 9 cases (0.6%) with mental illness.

There were significant differences in the percentage of hypertension, heart disease, diabetes, cerebral infarction, pulmonary disease and Alzheimer’s disease among elderly burns of different age groups (*P* < 0.05), while there were no significant differences in other pre-injury diseases. Among them, the percentages of hypertension, heart disease, cerebral infarction, pulmonary diseases and Alzheimer’s disease were significantly higher in the oldest geriatric group than in the younger group. In addition, the percentage of heart disease and cerebral infarction in the oldest geriatric group was significantly higher than that in the middle geriatric group, and the percentage of diabetes in the middle geriatric group was significantly higher than that in the oldest geriatric group (Table 3).

**Table 3:** Comparison of pre-injury diseases stratified per age category

Age categories(years)	Total (*N* = 1429)	60–69 (*n* = 796)	70–79 (*n* = 406)	80+ (*n* = 227)	*x*^*2*^	*P* value
Hypertension	512 (38.5%)	252 (31.7%)	153 (37.7%)	107 (47.1%)^a^	19.254	< 0.001
Heart disease	170 (11.9%)	71 (8.9%)	49 (12.1%)^f^	50 (22.0%)^b^	28.966	< 0.001
Diabetes	199 (13.9%)	104 (13.1%)	71 (17.5%)^g^	24 (10.6%)	6.918	0.031
Cerebral infarction	123 (8.6%)	51 (6.4%)	34 (8.4%)^h^	38 (16.7%)^c^	24.013	< 0.001
Pulmonary disease	51 (3.6%)	21 (2.6%)	14 (3.4%)	16 (7.0%)^d^	10.006	0.007
Alzheimer’s disease	30 (2.1%)	8 (1.0%)	10 (2.5%)	12 (5.3%)^e^	16.117	< 0.001

Bonferroni correction was applied to the* P* values for multiple comparisons between groups

^a^ 60-69y vs 80 + y, *P* < 0.001; ^b^ 60-69y vs 80 + y, *P* < 0.001; ^c^ 60-69y vs 80 + y, *P* < 0.001; ^d^ 60-69y vs 80 + y, *P* = 0.006; ^e^ 60-69y vs 80 + y, *P* < 0.001; ^f^ 70-79y vs 80 + y, *P* = 0.003; ^g^ 70-79y vs 80 + y, *P* = 0.019; ^h^ 70-79y vs 80 + y, *P* = 0.003

The mortality of the elderly burns with pulmonary disease (10.6%), Alzheimer’s disease (20.0%), and cancer (16.7%) was significantly higher than that of the patients without corresponding diseases (2.4%; 2.3%; 2.5%, respectively) (*P* = 0.003; *P* < 0.001; *P* = 0.039, respectively).

There were 90 patients (6.3%) with inhalation injury and 70 patients (4.9%) with shock on admission. The median (Q1-Q3) burn area of elderly burns with inhalation injury [28 (12–60)% TBSA] was significantly higher than that of patients without inhalation injury [7 (2–13)% TBSA] (*Z* = 10.502, *P* < 0.001). Elderly burns with shock on admission had significantly higher median (Q1-Q3) burn area [48 (35–70)% TBSA] than patients without shock [7 (2–13)% TBSA] (*Z* = 12.829, *P* < 0.001).

### Surgery

There were 2448 patients (95.8% of the total elderly burns) with information about whether to have surgery or not, among which 915 patients (37.4%) had undergone surgery during hospitalization, the average number of surgeries was 1.4 ± 1.1 and median of it was 1 (Q1-Q3, 1–2), and the average surgery cost was 9986 ± 12,842 CNY and median of it was 5530 (Q1-Q3, 2820–11,510) CNY. Among the elderly burns undergoing surgery, there were 688 cases (75.2%) of skin grafting, 125 cases (13.7%) of flap operation, 50 cases (5.5%) of amputation, and 35 (3.8%) cases of negative pressure sealing drainage.

There was a significant difference in the percentage of elderly burns undergoing surgery with different etiologies (x^*2*^ = 182.988, *P* < 0.001). The proportion of surgery from high to low was as follows: 74.6% of medical-related burns (44 cases), 74.1% of electrical contact burns (100 cases), 71.4% of hot crash injury (5 cases), 61.9% of low-thermal-burns (52 cases), 60.3% of contact burns with hot objects (38 cases), 46.2% of chemical burns (24 cases), 33.6% of scalds (338 cases), 30.6% of flame burns (311 cases) and 12.0% of electrical flash burns (3 cases).

### Length of stay and hospital cost

After excluding patients who were not cured or gave up treatment, there were 2231 patients (87.4% of the total) with hospital stay information, with a mean (SD) and median (Q1-Q3) of length of stay of 20.7 (27.9) days, and 14 (8–25) days, respectively.

There was a significant difference in the length of stay for different etiologies (x^*2*^ = 29.527, *P* < 0.001), and the median (Q1-Q3) length of stay of each etiology from high to low was: thermal crush injury (22 days, 19–27 days), electrical contact burns (17 days, 10–33.5 days), flame burns (15 days, 8–27 days), scalds (13 days, 8–23 days), contact burns with hot objects (13 days, 7.5–21.5 days), low-thermal-burns (13 days, 7.8–18.3 days), electrical flash burns (12 days, 7–16 days), medical-related burns (11 days, 7–17 days) and chemical burns (10 days, 5–15 days). Multiple linear regression showed that the factors influencing the length of stay of the elderly burns in this study included burn area (standard coefficient = 0.210, *P* < 0.001), number of surgery (standard coefficient = 0.418, *P* < 0.001).

After excluding patients who were not cured or gave up treatment, there were 1269 patients (46.7% of the total elderly burns) who had hospital cost information, with an average hospital cost of 31,243 ± 78,183 CNY and median of 10,410 (Q1-Q3, 5798–23,942) CNY, and an average hospital cost of per % TBSA of 8056 ± 44,201 CNY and median of 2137 (Q1-Q3, 891–5971) CNY.

**Fig. 5:**
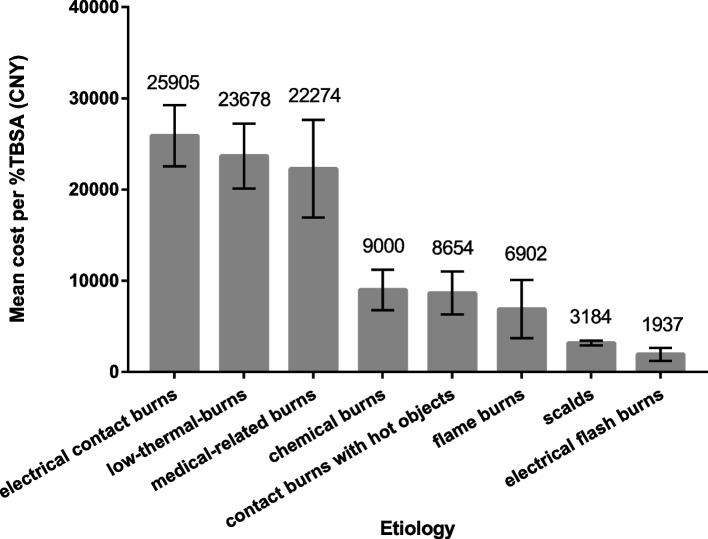
Comparison of mean per % TBSA cost of different etiologies

There was a significant difference in the average hospital cost of per %TBSA among elderly burns with different etiologies (*x*^*2*^ = 345.428, *P* < 0.001). The mean (SD) and median (Q1-Q3) hospital cost of each etiology from high to low was: electrical contact burns with 25,905 ± 28,045 CNY, 16,304 (8014–29,030) CNY, low-thermal-burns with 23,678 ± 30,662 CNY, 11,172 (4390–34,381) CNY, medical-related burns with 22,274 ± 38,951 CNY, 13,564 (8107- 24,865) CNY, contact burns with hot objects with 8654 ± 12,869 CNY, 4435 (1564–10,657) CNY, chemical burns with 9000 ± 12,919 CNY, 3755 (1964–10,449) CNY, flame burns with 3184 ± 5780 CNY, 1480 (763–3395) CNY, scalds with 6902 ± 68,673 CNY, 1427(831–3968) CNY, and electrical flash burns with 1937 ± 3469 CNY, 948 (682–1529) CNY (Fig. 5).

Multiple linear regression showed that the factors affecting the hospital cost of the elderly burns in this study included burn area, length of stay, surgery, inhalation injury, number of pre-injury diseases, and etiology (Table 4).

**Table 4:** Multivariate linear regression analysis of factors influencing hospital cost

	B	S.E	Beta	95% CI		*t*	*P* value
				Lower	Upper		
TBSA	2709.457	116.022	0.498	2481.838	2937.077	23.353	< 0.001
Length of stay	867.364	49.131	0.360	770.977	963.751	17.654	< 0.001
Surgery	26,251.054	3185.291	0.166	20,001.968	32,500.141	8.241	< 0.001
Inhalation injury	17,901.488	7577.727	0.048	3035.066	32,767.909	2.362	0.018
Number of Pre-injury diseases	3380.895	1387.340	0.047	659.131	6102.658	2.437	0.015
Etiology	1582.376	713.898	0.044	181.810	2982.943	2.217	0.027

Age,TBSA, length of stay, and number of pre-injury diseases were continuous variables; The categorical variables were included: Gender (male = 1, female = 0); depth of burn (partial-thickness = 0, full-thickness = 1); Etiology (scalds = 0, flame burns = 1, electric flash burns = 2, contact burns with hot objects = 3, chemical burns = 4, medical-related burns = 5, low-thermal-burns = 6, electrical contact burns = 7); Inhalation injury (No = 0, yes = 1); Surgery(No = 0, yes = 1); Shock on admission (No = 0, yes = 1). The value of the adjusted R2 for the regression was 0.742

*B* Nonstandard coefficients, *S.E*. Standard error, *Beta* Standard coefficients, *CI* Confidence interval

### Treatment outcomes

**Fig. 6:**
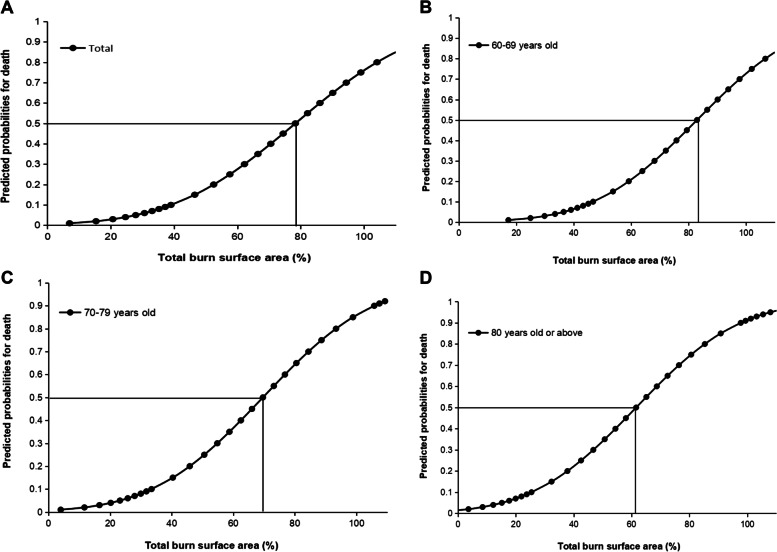
The lethal area 50% (LA_50_) for elderly burns. **A** LA_50_ for total admitted elderly burns. **B**. LA_50_ for 60–69 years old burns. **C**. LA_50_ for 70–79 years old burns. **D**. LA_50_ for 80 years old or above burns

After excluding those patients who were transferred to outpatient or other hospitals for treatment (183 cases) or gave up treatment (34 cases) and missing data of treatment outcome (106 cases), there were 2231 patients (87.4% of the total elderly burns) with treatment outcome information, including 2163 patients (97.0%) cured, and 68 patients (3.0%) died. The lethal area 50% (LA_50_) for total admitted elderly burns was 78.3% TBSA (95% confidence interval [CI] = 69.8 ~ 89.9% TBSA). The results showed that the age of elderly burns was negatively correlated with LA_50_. The LA_50_ was 82.9% TBSA (95% CI = 73.3 ~ 95.8% TBSA) in the young geriatric group, 69.6% TBSA (95% CI = 59.2 ~ 83.5% TBSA) in the middle geriatric group and 61.6% TBSA (95% CI = 50.9 ~ 75.3% TBSA) in the oldest geriatric group (Fig. 6). Multivariate logistic regression showed that the risk factors for mortality were TBSA, inhalation injury, pulmonary disease and Alzheimer’s disease (Table 5).

**Table 5:** Multivariate logistic regression analysis of risk factors of mortality (*n* = 2231)

	R	Crude		Adjusted for case mix		*P* value
		OR	95% CI	OR	95% CI	
Age	0.050*	1.038	1.009–1.067	1.019	0.969–1.072	0.464
TBSA group	0.243*	-	-	-	-	< 0.001
Small area = reference						
Medium area	-	3.657	1.507–8.876	6.444	1.559–26.640	0.010
Large area	-	10.841	3.848–30.539	21.624	4.139–112.975	< 0.001
Extra-large area	-	97.040	42.309–222.570	161.336	34.590–752.507	< 0.001
Inhalation injury	0.367*	27.867	13.700–56.684	5.308	1.959–14.384	0.001
Pulmonary disease	0.097*	4.906	1.879–20.332	11.307	2.623–48.740	0.001
Alzheimer’s disease	0.152*	10.491	0.107–6.776	24.824	5.707–107.976	< 0.001

Total burn surface area (TBSA), small area (< 10%TBSA), medium area (10–29%TBSA), large area (30–49%TBSA), extra-large area (≥ 50%TBSA)

*R* Spearman contingency coefficient, *OR* Odds ratio, *CI* Confidence interval

^*^means *P* value < 0.05. The ROC AUC value (with 95% CI) for the multivariable regression was 0.914 (0.855–0.974) (*P* < 0.001)

There were thirty-four elderly burns who gave up treatment (1.3% of the total elderly burns), with an average age of 74.6 ± 9.4 years, a median (Q1-Q3) burn area of 41.0 (32.5–67.5)% TBSA, a median (Q1-Q3) full-thickness burn area of 31.3 (10.3–40.8)% TBSA and a median (Q1-Q3) length of stay of 1 (1–3.3) day. Moreover, there were 18 males (52.9%) and 16 females (47.1%) in the gave up elderly burns.

There were significant differences in the percentage of elderly burns who gave up treatment to the total number of elderly burns in different age groups (x^*2*^ = 7.937, *P* = 0.019). The were 11 cases (0.9%) in the young geriatric group and 12 cases (1.5%) in the middle geriatric group, and 11 cases (2.8%) in the elderly geriatric group, respectively.

There were significant differences in the percentage of elderly burns who gave up treatment to the total number of elderly burns in different burn area groups (*x*^*2*^ = 85.574, *P* < 0.001).The were 2 cases (0.2%) of small area burns, 6 cases (1.0%) of medium area burns, 13 cases (9.0%) of large area burns, and 13 cases (12.5%) of extra-large area burns.

The percentage of elderly burns with full-thickness burns who gave up treatment (31 cases, 3.3%) was significantly higher than that of elderly burns with partial-thickness burns (1 case, 0.2%) (*x*^*2*^ = 14.209, *P* < 0.001).

## Discussion

Population aging has become one of the most important social trends in the twenty-first century, and China is striding towards an aging society. Due to China's vast territory, large population, huge regional differences in medical conditions and imperfect medical data statistical system, there is still a lack of epidemiological data on elderly burn patients in China, and most of the literature reports are single-center analysis of hospitalized elderly burn patients.

At present, the reported proportion of elderly burns in China has increased from 3.4% [15] (patients admitted from 1999 to 2004) or 3.8% [7] (patients admitted from 2003 to 2009) in the early stage, to 7.5% [5] (patients admitted from 2010 to 2016) in the recent stage. This study showed that the proportion of elderly burns has increased from 5.7% in 2004 to 8.6% in 2018, which was generally consistent with previous reports, but lower than that in developed countries such as the Netherlands [13] (10.0%, patients admitted from 2009 to 2015), the United States [10] (10.1%, patients discharged from 1997 to 2010). This may be related to the lack of medical resources and ability to pay for medical expenses in China, leading to more elderly burns being treated in outpatient clinics or even at home. In the past 15 years, the number of elderly burns had gradually increased in this study, which is consistent with the rising trend of elderly burns found in many studies [5, 14, 21]. Therefore, China and even the whole world should strengthen the capacity building of the prevention, first aid and medical treatment of the elderly burns in order to cope with the increasing number of elderly burn cases.

Due to various reasons such as physical function, living condition and social environment, the elderly burns have many different characteristics from burns in children and young and middle-aged people, and their disease development, treatment process and outcomes are also very different. Therefore, the study on the epidemiology of elderly burns is of great significance for the prevention, treatment and rehabilitation of burns in the elderly. This study showed that the majority of the elderly burns occurred at home, especially in the elderly aged 80 or over, with the proportion reaching 81.8%, which is similar to the previous report [12, 15]. Therefore, we suggest that the prevention of elderly burns should focus on improving the living environment of the elderly, for example, carry out the regular door-to-door maintenance of natural gas pipelines and electric wires by professional technicians, and install heating equipment for conditional families [22]. According to the experience of developed countries, smoke detection and alarm systems can effectively reduce the incidence of burns [21]. However, there are few Chinese families installed domestic fire-fighting equipment.

The most common causes of elderly burns are flame burns and scalds, which account for more than 80% of the total number of patients in this study. As a mega-city in central China, Wuhan has a hot and humid summer and cold winter climate, and the main heat source for residents in daily life is gas. Therefore, accidental indoor gas fire is a common cause of elderly burns. In addition, cooking at home, drinking hot soup or tea, and traditional bathing methods (such as bathing with hot water in a basin), are the main causes of elderly burns in this area. Elderly people who live alone and are slow to move, while lack of care and have some dangerous habits such as smoking in bed, also have an increased risk of burns. In addition, there is also an obvious age difference in elderly burns. Contact burns with hot objects are common in middle-aged and elderly people (70 years or over), while electrical burns are more common in young male elderly people (60–69 years old) who have more chance to do electric contacting jobs or fishing under high-voltage electrical power lines. However, the elderly often lack correct on-site emergency management after burns. Most of them (78.4%) did not receive any emergency management after burns. Only 8.5% of them were washed with cold water after burns, but the washing time was mostly less than 10 min. In recent years, the Chinese government has increased social support funds for the elderly. For example, in communities with a large number of elderly people, the 15-min life circle including medical services is established by government, so that the elderly can get medical treatment in time. What’s more, emergency call buttons in both the room and the toilet are set which could directly link the community, and safety hazards are regularly checked to ensure a safe living environment for the elderly. These are positive preventive measures to deal with accidental burns in the elderly.

Based on the time interval between injury and admission, nearly 40% of elderly burns went to hospital more than 24 h, and the older the age, the longer the time of admission, which may be related to the elderly's dependence on help, difficulty in seeking medical treatment and lower desire for medical treatment. Some elderly people use some folk remedies to treat wounds at home after burns, such as toothpaste, eggs, soy sauce, vinegar, salt, which increased the risk of wound contamination and infection. According to the above risk factors and characteristics of elderly burns, taking corresponding preventive and educational measures can help reduce the incidence rate of elderly burns, and avoid or reduce the risk of wound deepening, pollution and infection caused by improper post-injury treatment.

Since elderly burns are often accompanied by pre-injury diseases before injury [5, 6, 23], most patients in this study had one or more pre-injury diseases. Previous studies have shown that age is positively associated with heart disease and cerebral infarction. In this study, the mortality rate of older burn patients with pulmonary disease, Alzheimer’s disease or cancer were higher than the others. In this study, the mortality rate of elderly burns was 3%, which is lower than other reports (8%-14.9%) [15, 21]. The LA_50_ of elderly burns in this group was 78.3% TBSA, which is much higher than that of high-income countries (about 30% TBSA) [24], as well as low-income countries (45.3% TBSA) [25]. Our burn center is the largest burn center in central China, with large number of annual inpatients and outpatients. During the over half century history of this burn center, rich experiences have been accumulated for saving the burn victims, which lead to the high level of treatment. As for the elderly burns in our burn center, effective strategy of treatments include good initial fluid resuscitation, relative looser criteria of tracheotomy and mechanical ventilation for inhalation injury, timely and effective repair of deep partial- and full-thickness wounds, appropriate anti-biotic using and nutrition treatment, etc. For extensive deep partial- and full-thickness burns, it is utmost important to timely complete escharectomy and cover the wounds. Our strategy is try to finish the procedure within three to seven days post-burn by one or two operations even for elderly patients with full-thickness burns over 80–90%TBSA, on the basis of a stable resuscitation. Mostly we cover the extensive wounds by large sheet of allografts with auto-microskins [20], with the procedure rather simple and area of donor site very small, which is utmost important for extensive deep burns, especial for elderly burns. The physical function of the old people were weak, and usually accompanied by several chronic diseases, so the mortality rate of elderly burns is relatively high. On the basis of reasonable and effective comprehensive therapies, our strategies for repairing extensive deep burn wounds can timely and effectively cover the wounds, greatly provide support for the systemic therapy, and significantly improve the survival rate of elderly burn patients.

The treatment of elderly burns involves many problems such as medical aid system and social ethics [6, 13]. In this study, 34 patients (1.3%) gave up treatment by themselves or their families. Patient age and burn area were positively correlated with the proportion of abandoned treatment. Severe trauma, long and painful treatment process, and unbearable economic burden often make elderly burns and their families lose confidence in treatment, leading to some patients to give up treatment. Nevertheless, with the gradual improvement of China's medical security system and the full coverage of medical insurance for urban and rural residents, the number of elderly burns who give up treatment for economic reasons has gradually decreased in recent years. Nevertheless, it is still very important to provide adequate psychological, affectional and financial supports to elderly people with severe burns to prevent them from giving up. Medical staff should provide correct and professional information support and guidance to patients and their families, so as to reduce their anxiety, and help those with financial difficulties to obtain funding through community, government, charity organization, non-governmental organization, online social platform and other channels, which will help reduce the abandonment of treatment.

As the biggest burn center in central China, our patients come from Wuhan and the surrounding provinces. This study may represent not only epidemiological trends and characteristics of elderly burns in the provincial capital city and the whole province, but also more or less that of central China. Results from this study indicate an urgently need to pay attention for prevention, correct first aid and treatment of elderly burns by the government, medical systems and social organizations. However, some missing of detailed injury information, accurate pre-hospital emergency management data, and lack of enough follow-up are the limitations of this research. Further future prospective and multi-center study are necessary to get better understanding of the elderly burns.

## Conclusion

Geriatric burns was still common and even increasing in central China, with flame burns and scalds the most common causes, majority of whom injured at home and often had problems such as few cooling treatment, improper emergency management and delayed admission. TBSA, etiology, pre-injury diseases and inhalation injury were the risk factors of length of stay, hospital cost and treatment outcomes.

## Data Availability

The data sets used for the analysis in the current study are available from the corresponding author on reasonable request.

## Abbreviations

TBSA: Total body surface area
LOS: Length of stay
SD: Standard deviation
Q1-Q3: 25-75th percentile
LA_50_: Lethal area 50
